# Update on Cardioprotective Strategies for STEMI: Focus on Supersaturated Oxygen Delivery

**DOI:** 10.1016/j.jacbts.2021.07.011

**Published:** 2021-10-27

**Authors:** Robert A. Kloner, Jeffrey L. Creech, Gregg W. Stone, William W. O’Neill, Daniel Burkhoff, J. Richard Spears

**Affiliations:** a Department of Medicine and Division of Cardiovascular Medicine, Keck School of Medicine at University of Southern California, Los Angeles, California, USA; b Huntington Medical Research Institutes, Pasadena, California, USA; c ZOLL TherOx, Irvine, California, USA; d The Zena and Michael A. Wiener Cardiovascular Institute, Department of Medicine, Division of Cardiology, Icahn School of Medicine at Mount Sinai, New York, New York, USA; e Cardiovascular Research Foundation, New York, New York, USA; f Department of Internal Medicine, Division of Cardiology, Henry Ford Hospital System, Detroit, Michigan, USA; g Columbia University Medical Center, New York, New York, USA; h Department of Cardiovascular Medicine, Beaumont Systems, Royal Oak, Michigan, USA

**Keywords:** LV function, LV remodeling, myocardial infarct size reduction, ST-segment elevation myocardial infarction, supersaturated oxygen, AMI, acute myocardial infarction, CMR, cardiac magnetic resonance, FDA, Food and Drug Administration, HF, heart failure, LAD, left anterior descending coronary artery, LM, left main coronary artery, LV, left ventricular, LVEF, left ventricular ejection fraction, MI, myocardial infarction, NACE, net adverse clinical events, Pao_2_, partial pressure of oxygen, PCI, percutaneous coronary intervention, SPECT, single-photon emission computed tomography, SSO_2_, supersaturated oxygen, STEMI, ST-segment elevation myocardial infarction, TIMI, Thrombolysis In Myocardial Infarction, TVR, target vessel revascularization

## Abstract

Despite the fact that door-to-balloon times have been greatly reduced, the rates of death and the incidence of heart failure in patients with ST-segment elevation myocardial infarction (MI) have plateaued. There is still an unmet need to further reduce MI size in the reperfusion era. Most adjunctive therapies to enhance myocardial salvage have failed, but some have shown promise. Currently, the only adjunctive therapy in a pivotal trial that has demonstrated reductions in infarct size is localized delivery of supersaturated oxygen (SSO_2_) therapy. This review provides background on prior infarct size reduction efforts. The authors describe the preclinical data that shows the effectiveness of SSO_2_ in reducing MI size, improving regional myocardial blood flow and cardiac function, and reducing adverse left ventricular remodeling—presumably by reducing patchy areas of residual ischemia within the reperfused risk zone. Potential mechanisms by which SSO_2_ is beneficial are described, including the delivery of high levels of dissolved oxygen through plasma to ischemic, but viable, vascular and myocardial cells, thus allowing their survival and function. The authors then describe the SSO_2_ clinical trials, demonstrating that in patients with anterior ST-segment elevation MI, SSO_2_ therapy safely and effectively reduces infarct size, improves cardiac function, and reduces adverse left ventricular remodeling.

## Highlights

• Beyond restoring myocardial perfusion, there is an unmet need to further reduce the size of myocardial infarctions; only 1 therapy is FDA-approved to specifically treat ischemic myocardium as an adjunct to reperfusion therapy.
• Here, we present the basic science and clinical studies showing that treatment with supersaturated oxygen following mechanical reperfusion therapy in patients with large anterior myocardial infarctions improves myocardial perfusion and reduces infarct size.
• Early clinical studies also show that supersaturated oxygen improves left ventricular function and reduces adverse left ventricular remodeling in patients with large anterior myocardial infarctions.

In patients with ST-segment elevation myocardial infarction (STEMI) undergoing mechanical reperfusion therapy, numerous adjunctive approaches have been developed to further reduce the extent of myonecrosis (1,2). The purpose of the present review is to describe these attempts (many of which have ultimately failed), and to focus on the relatively new therapy of delivering supersaturated oxygen (SSO_2_) to ischemic/reperfused myocardium. Our review includes analysis of preclinical studies as well as completed clinical trials with SSO_2_ (Central Illustration).

**Central Illustration:**
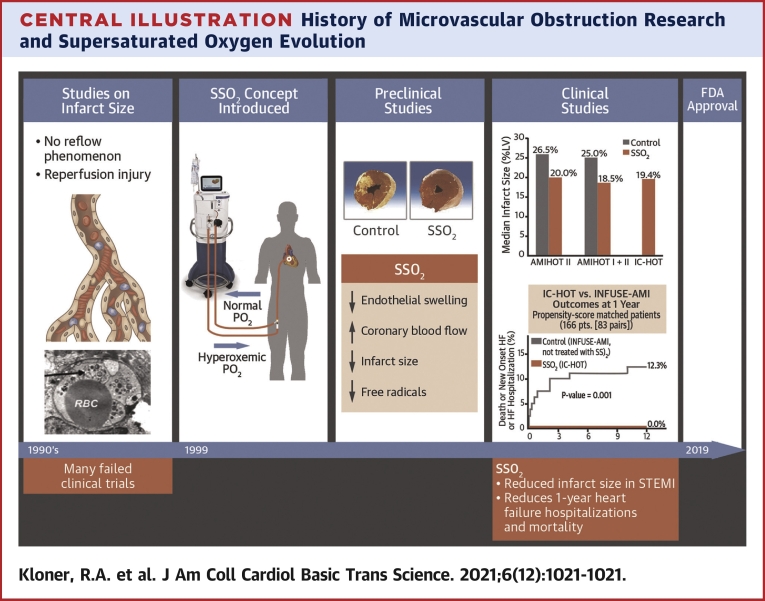
History of Microvascular Obstruction Research and Supersaturated Oxygen Evolution Timeline of microvascular obstruction (MVO) research/understanding and supersaturated oxygen (SSO_2_) therapy concept development, clinical study, and approval. **(First panel, Studies on infarct size)(Top)** Medical illustration depicting MVO post-STEMI. **(Bottom)** Electron microscopic view of swollen endothelial cell **(arrow)** post-infarct in porcine model (46). There were many failed clinical trials with a variety of treatments that attempted to further reduce infarct size above and beyond reperfusion therapy; **(Second panel, SSO_2_ Concept Introduced)** SSO_2_ concept drawing showing administration of this therapy in the catheterization laboratory; **(Third panel, Preclinical Studies)(Top)** Porcine transverse myocardial slices post- infarct in control pig **(left)** showing large infarct and in treated pig **(right)** showing minimal infarction (45). **(Bottom)** Preclinical studies showed several beneficial effects of SSO_2_ therapy; **(Fourth Panel, Clinical Studies) (Top)** Summary of consistent infarct size reduction trials conducted by TherOx, Inc (40,50,55); **(Bottom)** one-year propensity-matched IC-HOT data demonstrating no death, no new onset heart failure (HF), and no HF hospitalizations with SSO_2_ therapy; chart supplied by G.W. Stone from data in Chen et al (57); **(Fifth Panel, FDA Approval, 2019).** FDA = Food and Drug Administration; IC-HOT = Intracoronary Hyperoxemic Oxygen Therapy study; INFUSE-AMI = The INFUSE-Anterior Myocardial Infarction (AMI) study; RBC = red blood cell; STEMI = ST-segment elevation myocardial infarction.

## Background

In 1971, Peter Maroko and Eugene Braunwald published a seminal paper establishing various factors that could influence myocardial infarction (MI) size (Maroko et al [3]). In 1977, Keith Reimer and Robert Jennings described the “march” of necrosis from the subendocardium to subepicardium over the course of 24 hours after coronary occlusion, and demonstrated that reperfusion could salvage ischemic, but viable, tissue in experimental models (Reimer et al [4]). To salvage substantial amounts of myocardium, reperfusion within 6 hours and preferably within 3 hours of coronary occlusion was essential, the time after which the wave front of necrosis extended to the outer wall of the ventricle.

Experimental studies confirmed that early reperfusion reduced infarct size (5), and clinical studies revealed that effectively reperfusing the infarcted artery as soon as possible and preventing reocclusion were the foundations to minimize infarct size and mortality. Stenting plus abciximab was shown to reduce infarct size compared to alteplase alone (6). Pharmacologic agents administered adjunctively with reperfusion therapy for STEMI include: 1) potent antiplatelet agents to prevent reocclusion after stent implantation; 2) statins, ezetimibe, and PCSK9 inhibitors to stabilize atherosclerotic plaque and reduce progression of atherosclerosis (7); and 3) pharmacologic agents to reduce adverse left ventricular (LV) remodeling post-infarction (eg, renin-angiotensin-aldosterone system inhibitors, beta-blockers, and possibly neprilysin inhibitors (8).

However, there is still an unmet need to further reduce the MI size using agents and therapies that directly protect the myocardium while opening the infarcted artery and keeping it patent. Despite reducing mean door-to-balloon times to <90 minutes (∼60 minutes at most major U.S. medical centers) and the routine use of optimal pharmacology, 20%-30% of acute MI (AMI) patients still develop heart failure (HF) within 1 year (9), and 30-day mortality rates in AMI patients >65 years of age are approximately 12% (10). Unfortunately, resulting infarct size is often substantial, despite timely myocardial reperfusion (11). Although 85%-90% of STEMI patients experience restored coronary flow (Thrombolysis In Myocardial Infarction [TIMI] flow grade 3) via primary percutaneous coronary intervention (PCI), it has been observed that microcirculatory perfusion and myocardial metabolism are not restored in ∼50% of cases (12,13).

Salvaging an additional 5% of the LV myocardium can have major beneficial effects on clinical outcomes, including reducing mortality and new-onset HF (11). Therapies beyond timely reperfusion to further reduce infarct size have been studied over several decades. Yet, although many such therapies have shown promise in the experimental laboratory, few have shown benefit in randomized clinical trials (14, 15, 16).

Many therapies that worked in experimental models failed when tested clinically (often referred to as the “graveyard” of therapies) (14, 15, 16). There are potential reasons for these failures as described in previous reviews (14, 15, 16).

One major mechanism of infarct size reduction targeted by many of these therapies is the prevention of reperfusion injury (ie, the mechanisms whereby the reperfusion process itself harms ischemic, but not yet necrosed, cardiomyocytes). Whereas some investigators claim that about one-half of all cell death during an infarct is caused by reperfusion, others are less convinced of the existence of reperfusion injury in humans or its substantial role in myonecrosis (17, 18, 19).

## Beyond the Graveyard: Promising Therapies to Reduce Infarct Size

Despite a host of negative studies, there are still a few approaches that have shown promise for further reducing MI size beyond epicardial reperfusion alone (Table 1). Some recent studies have also suggested that a few of these therapies may reduce adverse LV remodeling with or without acutely reducing MI size. First, in 2 separate multicenter randomized trials prolonged adenosine intravenous infusions resulted in a reduction of MI size in patients with anterior STEMI (20,21). It is important to note that in these studies, adenosine was initiated before reperfusion and included a lengthy (3-hour) infusion. Studies of boluses or short infusions using the intracoronary route for adenosine delivery have generally been negative. This is not surprising given that the half-life of adenosine is very short, so any benefit from a bolus therapy or short-term infusion would be short-lived. In the AMISTAD II trial (A Randomized, Double-Blinded, Placebo-Controlled Multicenter Trial of Adenosine as an Adjunct to Reperfusion in the Treatment of Acute Myocardial Infarction), patients who were reperfused within ∼3 hours and received a 3-hour intravenous adenosine infusion demonstrated better clinical outcomes than those who received a placebo (22).

**Table 1:** Studies of Adjunctive Therapies With Reperfusion That Show Some Positive Results

Type of Agent	Study/First Author, Year (Ref. #)	Outcome
Adenosine	AMISTAD I and II, 1999-2006 (20, 21, 22)	Reduced anterior MI size. With early (≤3 h) reperfusion improved clinical outcomes (AMISTAD II)
Therapeutic hypothermia	COOL MI, 2003 (24)	No overall difference in infarct size, clinical events. However, patients with anterior MI cooled <35 °C before PCI had smaller infarcts
Hyperoxemic reperfusion, SSO_2_	AMIHOT I + II, 2009, 2007 (40,50)	Patients with anterior MI reperfused <6 h had smaller infarcts: 25% control vs 18.5% SSO_2_
PiCSO	Egred et al, 2020 (28)	Smaller infarct size with PiCSO (14%) than historically matched controls from INFUSE-MI (21%)
RIC	Bøtker et al, 2010; Sloth et al, 2014; Hausenloy et al, 2019; Ikonomidis et al, 2021 (33, 34, 35, 36)	Botker/Sloth—RIC improved salvage index and long-term clinical outcomes. Hausenloy—(CONDI-2/ERIC-PPCI): Large study showed no effect on cardiac death/CHF. Note: low-risk controls Ikonomidis 2021—RIC reduced adverse LV remodeling
Mechanical post-conditioning	Staat et al, 2005; Traverse et al, 2019 (30,31)	Staat: Showed that post-conditioning at the time of PCI reduced infarct size. Traverse: No effect of mechanical post-conditioning on infarct size, but it did reduce adverse LV remodeling.
Chemical post-conditioning (cyclosporine)	Piot et al, 2008; Cung et al, 2015 (29,32)	Piot et al: Cyclosporine given with reperfusion reduced infarct size. Cung et al, in a larger study, failed to show a benefit of cyclosporine
LV unloading before reperfusion	Kapur et al, 2019 (26)	Delayed reperfusion for 30 min with LV unloading did not increase infarct size (13%) vs immediate reperfusion (15%)
Human atrial natriuretic peptide	Kitakaze et al, 2007 (37)	Human atrial natriuretic peptide reduced infarct size, but nicorandil did not
Exenitide (antidiabetic agent)	Lønborg et al, 2014 Roos et al, 2016 (38,39)	Lønborg study: Exenatide reduced infarct size whether patients were hyperglycemic or not. No effect on infarct size in the Roos study.

AMIHOT = Acute Myocardial Infarction With HyperOxemic Therapy study; AMISTAD I = Acute Myocardial Infarction STudy of ADenosine; AMISTAD II = A Randomized, Double-Blinded, Placebo-Controlled Multicenter Trial of Adenosine as an Adjunct to Reperfusion in the Treatment of Acute Myocardial Infarction; CHF = congestive heart failure; CONDI-2/ERIC-PPCI = Effect of Remote Ischaemic Conditioning on Clinical Outcomes in STEMI Patients Undergoing PPCI study; COOL MI = Cooling as an Adjunctive Therapy to Percutaneous Intervention in Patients With Acute Myocardial Infarction study; INFUSE-AMI = The INFUSE - Anterior Myocardial Infarction (AMI) study; LV = left ventricular; MI = myocardial infarction; PCI = percutaneous coronary intervention; PiCSO = pressure-controlled intermittent coronary sinus occlusion; RIC = remote ischemic conditioning; SSO_2_ = supersaturated oxygen.

Systemic hypothermia has been demonstrated in preclinical models to reduce infarct size (23). The pivotal COOL MI (Cooling as an Adjunctive Therapy to Percutaneous Intervention in Patients With Acute Myocardial Infarction) trial did not show such an effect for all patients, although infarct size reduction was observed in patients with anterior infarcts that were successfully cooled to <35 °C before primary PCI (24). However, a recent study of rapid intravascular cooling before reperfusion for acute STEMI did not demonstrate a reduction in infarct size, which may have been in part caused by an offsetting effect from greater delay in time to reperfusion (25).

LV unloading before reperfusion may reduce infarct size by decreasing oxygen demand, increasing oxygen supply, and activating cardioprotective mechanisms (26), which is being tested in an ongoing pivotal randomized trial (27). Pilot studies have suggested that pressure-controlled intermittent coronary sinus occlusion may reduce infarct size by redistributing coronary flow to watershed ischemic zones (28).

Studies on post-conditioning—a series of brief ischemia–reperfusion cycles induced after achieving patency of the infarct-related coronary artery—have been mixed, with early studies of mechanical or pharmacologic post-conditioning (eg, with cyclosporine A that also blocks opening of the mitochondrial permeability transition pore) showing enhanced myocardial salvage (29,30), but with later larger studies failing to show reduced infarct size (31) or improved clinical outcomes (32). However, 1 study did show that post-conditioning was associated with less adverse LV remodeling than in the placebo group (31), and most recently, a randomized trial in 270 patients reported that remote ischemic conditioning within 48 hours post-PCI reduced adverse LV remodeling (33). Remote ischemic conditioning (inducing ischemia in a remote tissue, such as a limb, before reperfusion) has also shown mixed results on MI size. An early study in 333 patients reported that this therapy reduced infarct size and improved long-term outcomes (34,35). A subsequent randomized trial in 5,401 patients failed to show any benefit (36). However, patients in this trial may have been low risk, because it was noted that the control group had an unusually low rate of adverse cardiovascular events. Other approaches include 1 2006 study reporting that atrial natriuretic peptide may modestly reduce infarct size and increase LV ejection fraction (LVEF) with no effect on mortality (37). Exenatide is a glucagon-like peptide-1 receptor agonist, which in 1 2014 study (38) increased salvage index in patients with MIs whether they were normoglycemic or hyperglycemic. However, a 2016 study failed to observe any reduction in infarct size with the addition of exenatide (39).

Hyperoxemic infusion of SSO_2_ therapy has shown promise as an adjunct to primary PCI. In the AMIHOT (Acute Myocardial Infarction with Hyperoxemia Therapy) I and II trials, patients with anterior wall STEMI who were reperfused within 6 hours had smaller LV infarcts with SSO_2_ therapy compared with controls (18.5% vs 25% of the total LV mass) (40). Following additional studies showing safety and consistent effectiveness, SSO_2_ therapy was Food and Drug Administration (FDA)-approved (April 2, 2019) “indicated for non-shock left anterior descending coronary artery (LAD) STEMI treated successfully with PCI within 6 hours of symptom onset.”

## SSO_2_ Therapy: Preclinical Studies

**Figure 1:**
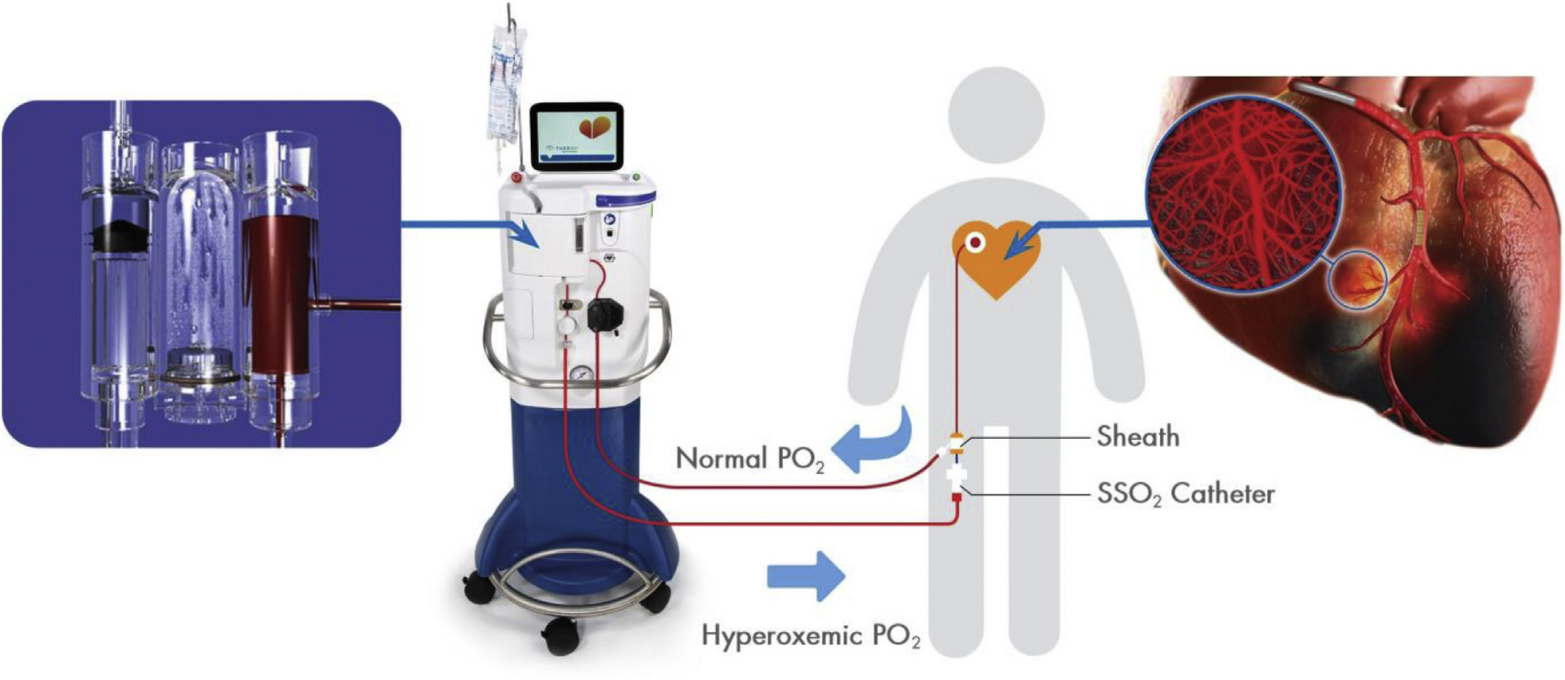
Basic SSO_2_ Delivery System The patient is administered a 60-minute infusion of supersaturated oxygenated blood at a flow rate of 100 ml/min with a PO_2_ (pO_2_) of 760-1,000 mm Hg into the left main coronary artery immediately following successful percutaneous coronary intervention. SSO_2_ = supersaturated oxygen.

**Figure 2:**
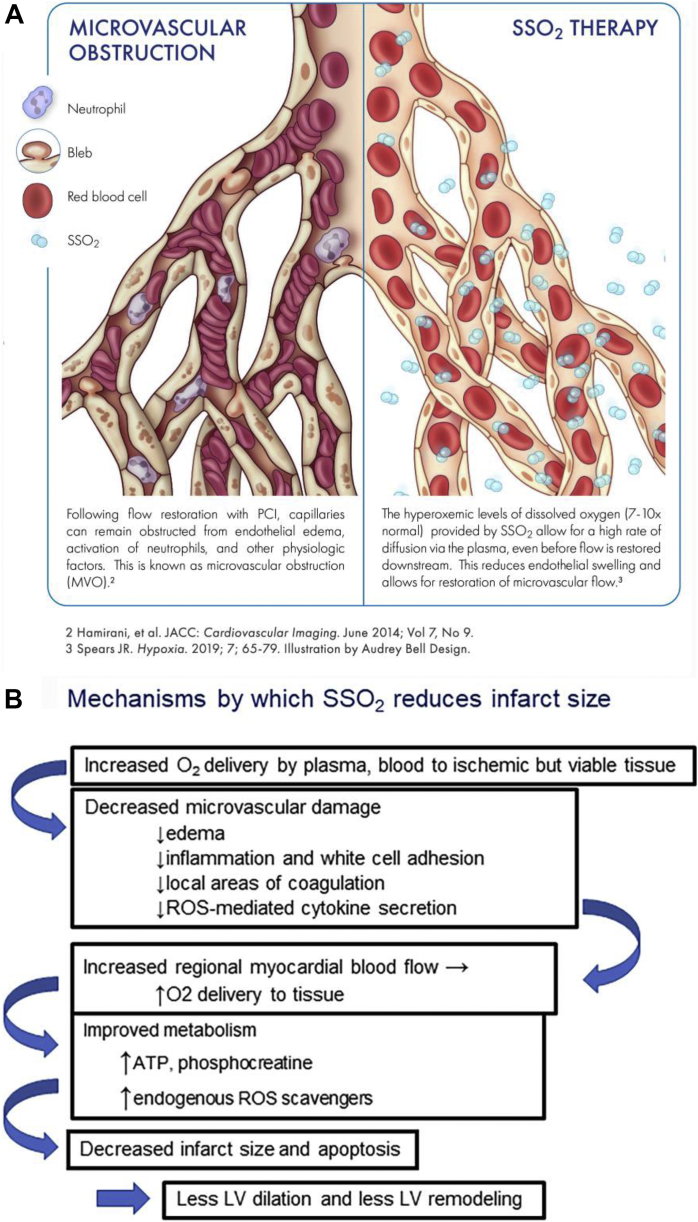
Proposed Mechanism How SSO_2_ Improves Endothelial Structure And Function, And Improves MVO Proposed mechanism of action regarding how supersaturated oxygen (SSO_2_) improves endothelial structure and function and improves microvascular obstruction (MVO; no-reflow/low reflow) post-establishment of patency of the infarct-related artery. **(A)** Schematic of microvascular obstruction with swollen endothelial cells and rouleaux formation within the small blood vessels. SSO_2_ therapy brings oxygen to the endothelial cell, presumably via diffusion through plasma, prevents the severe endothelial swelling and allows better perfusion. **(B)** Schematic of SSO_2_’s benefits early and late with reduction of not only infarct size but also adverse LV remodeling. ATP = adenosine triphosphate; LV = left ventricular; PCI = percutaneous coronary intervention; ROS = reactive oxygen species.

Spears (41) has reviewed the concept of patchy regions of residual microvascular ischemia that remain in the reperfused bed, even after establishing patency of the MI-related culprit epicardial coronary artery, and the mechanisms behind this phenomenon. Specifically, reperfusion is typically heterogeneous within the microvasculature, resulting in patchy areas of residual ischemia/hypoxia in the ischemic risk zone. Microvascular damage including endothelial cell swelling and microemboli may contribute. Although dense areas of no-reflow associated with lack of the fluorescent dye thioflavin S perfusion tend to be confined to areas of the myocardium already exhibiting necrosis (12), there may be areas of the myocardium where perfusion is reduced (stunned microvasculature) (42,43), but not low enough to exhibit anatomic perfusion defects. If there are focal areas of incomplete reperfusion caused by microvascular abnormalities in regions where myocardial cells are still viable, ongoing ischemia/hypoxia may lead to extension of myocardial cell death and increased infarct size. This phenomenon may be even more important in humans (compared with studies in experimental animal models of otherwise normal coronary arteries) caused by atherosclerotic and thrombotic microemboli that occur before and after PCI. Spears pioneered the delivery of SSO_2_ therapy to regions of microvascular ischemia to help counter the problem of ongoing cardiac cell death. He developed a catheter-based approach of delivering hyperbaric levels of oxygen to the ischemic myocardium without bubbles and was able to increase the PO_2_ (Pao_2_) of coronary blood perfusate to ∼900 mm Hg (Figure 1). Use of this SSO_2_ system was believed to increase oxygen delivery to both endothelial cells and myocardial tissue via oxygen diffusion in plasma. Improved oxygenation may preserve myocardial cells on the cusp of necrosis, because it limits microvascular damage during ischemia/reperfusion and increases regional myocardial blood flow, thereby further enhancing oxygen delivery to tissue by both diffusion and better microvascular blood flow (Figure 2).

**Figure 3:**
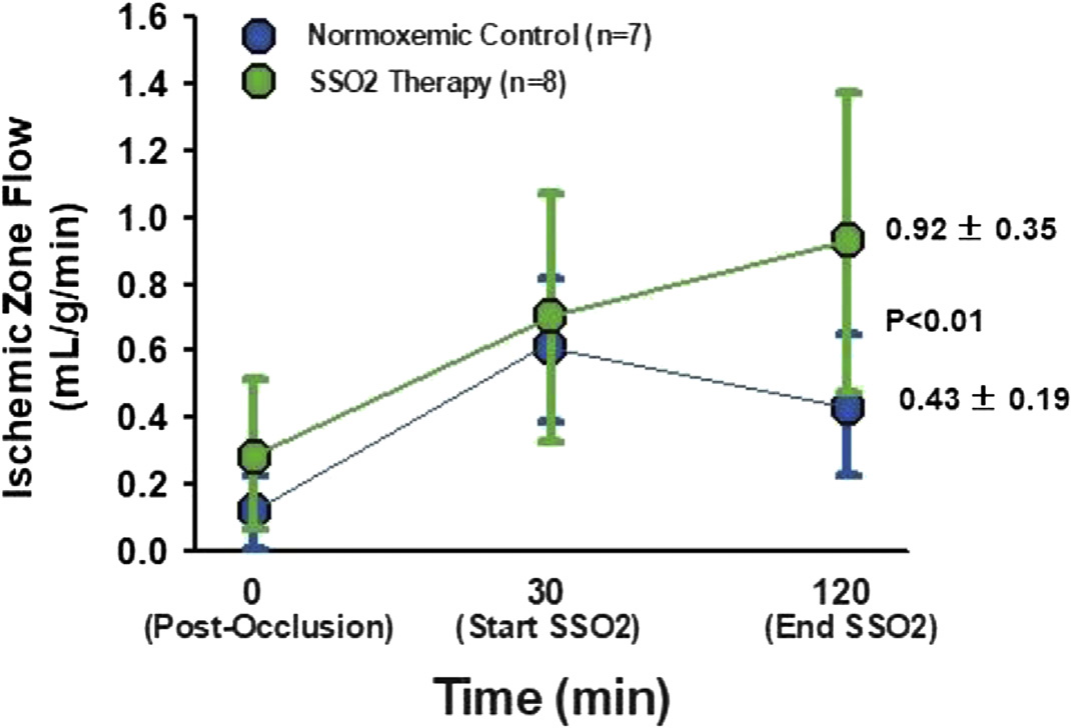
SSO_2_ in a Canine Model of Coronary Artery Occlusion and Reperfusion SSO_2_ given to a canine model of 90 minutes of coronary artery occlusion and reperfusion improved recovery of regional myocardial blood flow during reperfusion. From Spears et al (44); used with permission. SSO_2_ = supersaturated oxygen.

Several preclinical studies paved the way to clinical trials of SSO_2_ (previously referred to as aqueous oxygen) in patients. Spears et al (44) studied the effect of SSO_2_ intracoronary perfusion in a canine model inducing 90 minutes of coronary artery occlusion (balloon inflation) followed by reperfusion (balloon deflation). Intracoronary SSO_2_ therapy was started after 30 minutes of normoxemic autoreperfusion and was then continued for 90 minutes. Control groups received normoxemic reperfusion by either passive reperfusion or active reperfusion using a roller pump. Mean arterial Pao_2_ was 108 mm Hg in the normoxemic reperfusion groups and 530 mm Hg in the SSO_2_ group. Coronary artery balloon occlusion was associated with a mean acute decline in LVEF of 18%. In this study, a significant increase in LVEF (mean +17%) was observed in the SSO_2_ group at 90 minutes of reperfusion compared with normoxemic autoreperfusion (*P <* 0.05). In addition, SSO_2_ was associated with an improvement in LV regional wall motion abnormalities compared with normoxemic autoreperfusion. SSO_2_ improved ST-segment deviation and reduced the number of premature ventricular beats. At 120 minutes of reperfusion, regional myocardial blood flow, measured with radioactive microspheres, was 0.92 ml/g per minute in the previously ischemic zone of the SSO_2_-treated animals versus 0.43 ml/g per minute in the normoxemic autoreperfusion control group (*P <* 0.01) (Figure 3) (44). The investigators concluded that inadequate reperfusion occurs at the microvascular level despite patency of the epicardial infarct-related coronary artery. SSO_2_ improved LV function, electrocardiographic parameters of ischemia, and regional myocardial blood flow during the reperfusion phase (44).

**Figure 4:**
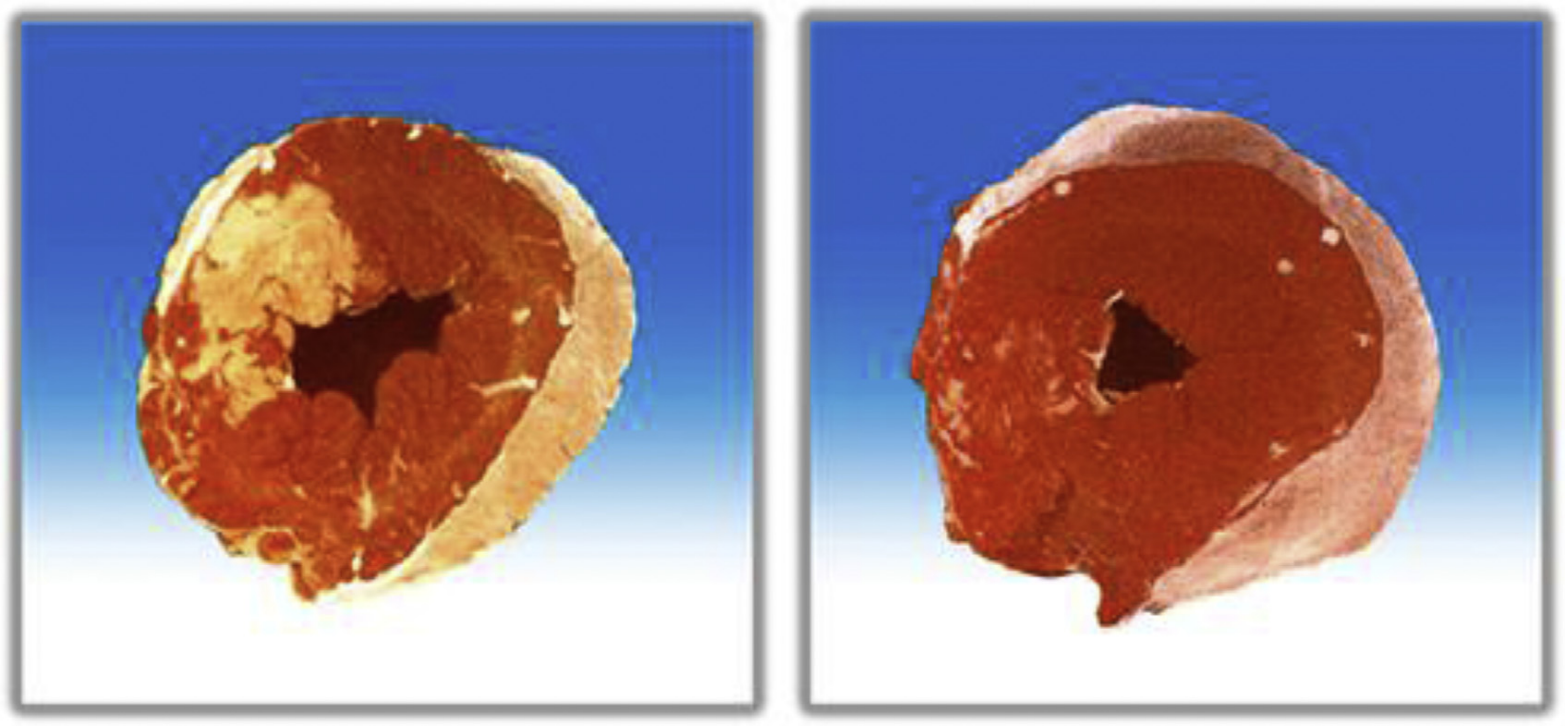
Representative Cross Sections of the LV of Normoxemic Control and SSO_2_ Therapy Pig Hearts Representative cross sections of the LV of pig hearts subjected to 60 minutes of coronary artery occlusion either treated with normoxemic control **(left)** or SSO_2_ therapy during reperfusion **(right)**. Heart slices were incubated in triphenyl-tetrazolium chloride. The infarct appears as a white area, whereas noninfarcted, viable tissue appears red. SSO_2_ therapy significantly reduced myocardial infarct size in this study. Original (unpublished) data from the study of Spears JR, Henney C, Prcevski P, et al. Aqueous oxygen hyperbaric reperfusion in a porcine model of myocardial infarction. *J Invasive Cardiol.* 2002;14:160-166. Abbreviations as in Figure 2.

**Figure 5:**
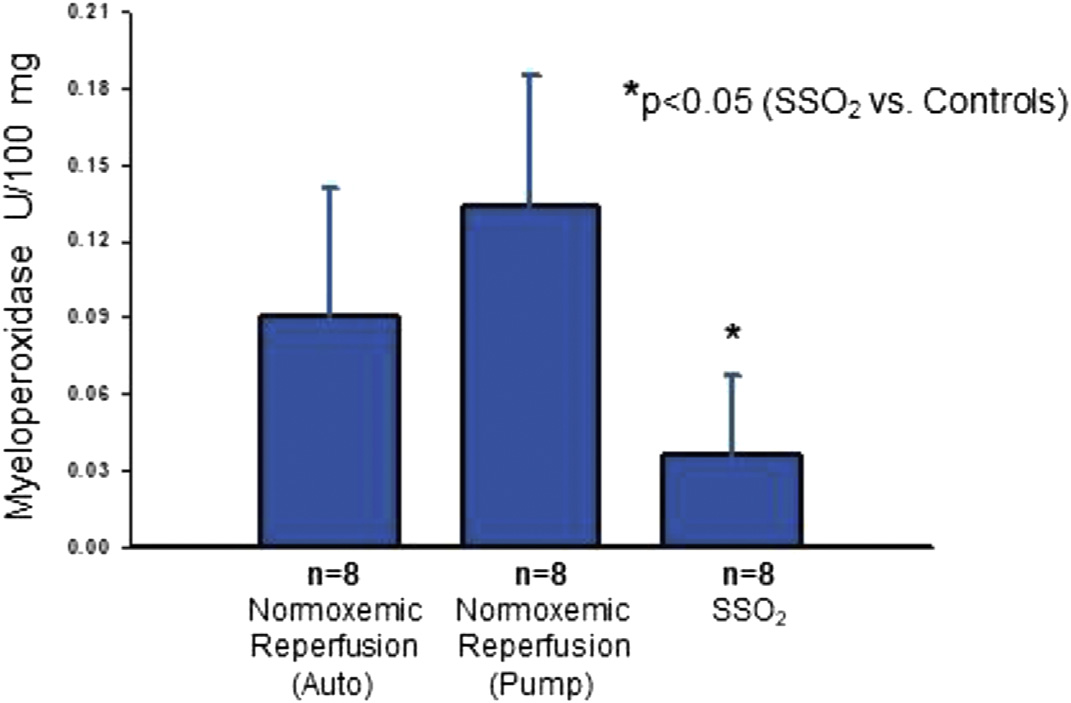
Myeloperoxidase Levels in Porcine Myocardium Myeloperoxidase levels in porcine myocardium at 3 hours of reperfusion from the studies described in Figure 4. The results suggest that SSO_2_ reduced oxygen free radical damage associated with ischemia/reperfusion in this experimental porcine model of coronary artery occlusion/reperfusion. From Spears JR, Henney C, Prcevski P, et al. Aqueous oxygen hyperbaric reperfusion in a porcine model of myocardial infarction. *J Invasive Cardiol.* 2002;14:160-166; reprinted with permission.

In a separate study, Spears et al (45) induced a 60-minute balloon occlusion in the LAD of pigs, followed by 15 minutes of normoxemic autoperfusion, then 90 minutes of intracoronary hyperoxemic perfusion (mean Pao_2_ of 834 mm Hg) versus several control groups, including continuous normoxemic autoperfusion and normoxemic reperfusion (using an active perfusion system similar to that used with SSO_2_ but without the SSO_2_). Reperfusion was continued for a total of 195 minutes. Serial LV angiograms were performed to assess LV function in this porcine model. Area at risk was determined by reoccluding the coronary artery and injecting blue dye into the left atrium. Following euthanasia, the hearts were sliced from apex to base and the transverse slices were incubated in triphenyl-tetrazolium chloride, which stains viable cells red, whereas infarcted myocardium appears unstained (white/yellow). Hyperoxemic reperfusion was associated with a higher LVEF (mean 62%) after 90 minutes of treatment compared with values during the 15-minute normoxemic autoreperfusion period in the same animals (mean 51%). Hyperoxemic reperfusion was associated with a higher LVEF (62% at 105 minutes and 60% at 165 minutes) than in the normoxemic groups at 105 minutes (autoreperfusion 45%, normoxemic reperfusion 48%) and 165 minutes (autoreperfusion 45%, normoxemic reperfusion 43%). Myocardial infarct size, expressed as percent area of necrosis divided by area at risk, was smaller in the hyperoxemic perfusion group (<20% of the LV) compared to the other control groups (between ∼70%-80% of the LV) (Figure 4). Hemorrhage score and myeloperoxidase levels (Figure 5) were also lower in the hyperoxemic perfusion group. The investigators concluded that intracoronary hyperbaric oxygen reperfusion reduced MI size and improved LV function.

**Figure 6:**
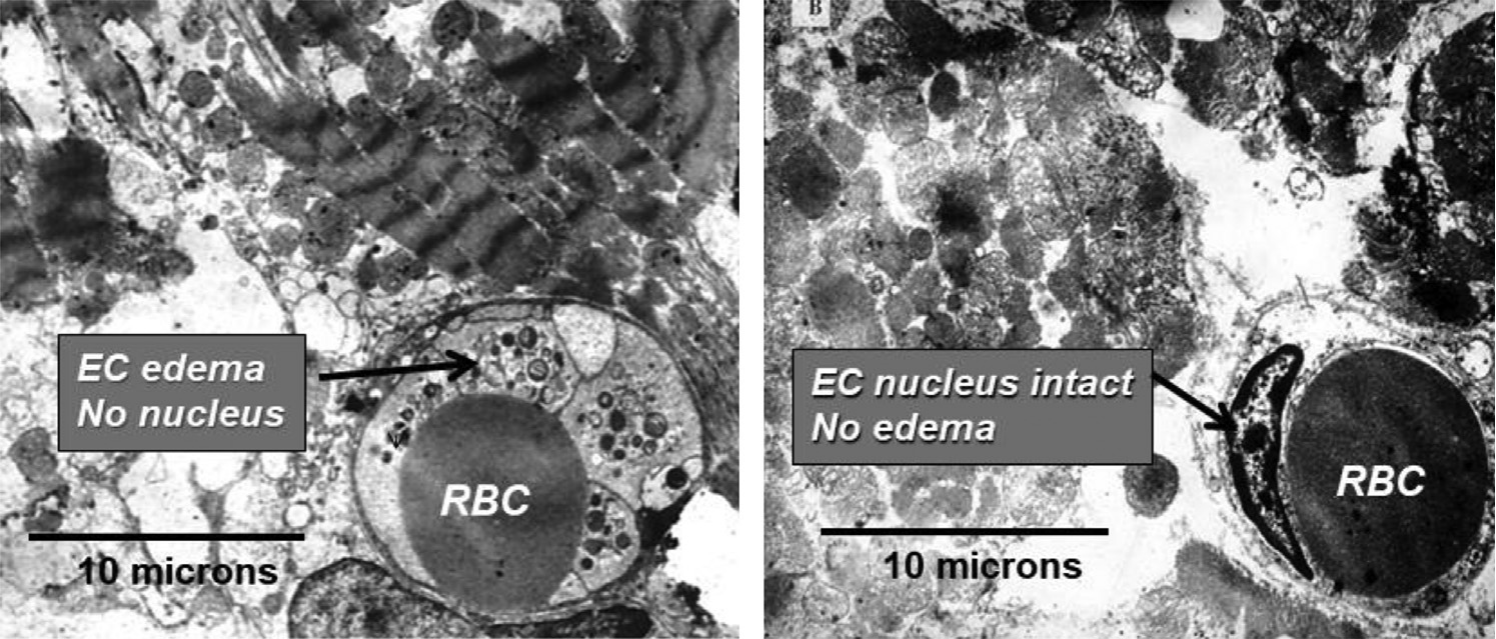
Electron Micrographs of Porcine Myocardium in Control and SSO_2_-Treated Pigs Electron micrographs of porcine myocardium at 3 hours of reperfusion after 1 hour of occlusion in a control pig **(left)** and SSO_2_-treated pig **(right)**. Note the endothelial swelling in the control group and the lack of endothelial swelling and preservation of the endothelial nucleus in the SSO_2_-treated group. This figure originally appeared in Bartorelli et al (46). Permission received from Springer Nature. EC = endothelial cell; RBC = red blood cell.

The findings regarding reduced myeloperoxidase levels, a marker of oxygen free radical tissue damage, may be especially important. Infusing hyperoxemic blood during reperfusion could theoretically exacerbate the burst of reactive oxygen species, which are known to generate upon reperfusion of previously ischemic tissue. It stands to reason that expected myeloperoxidase levels would be higher with hyperoxemic reperfusion. However, just the opposite occurred, suggesting that this therapy actually reduced reactive oxygen radical damage. This is a reassuring finding, consistent with the observed salutary benefits on infarct size. Another important finding from this study regarded differences in the ultrastructure of the myocardium. In the control group, endothelial edema and loss of endothelial cell nuclei were observed. Intracoronary hyperoxemic perfusion was associated with less endothelial cell edema and preserved endothelial nuclei (Figure 6). SSO_2_ treatment also prevented myocardial apoptosis (41,46).

Kantor et al (47) reported similar observations from a porcine model. These investigators induced a 60-minute coronary occlusion followed by randomization to reperfusion with selective 60 minutes of SSO_2_ infusion (Pao_2_ of 622 mm Hg) versus normoxemic autoperfusion. Echocardiographic assessment showed better LV function in the hyperoxemic group at 1 and 2 hours after randomization. There was a reduction in infarct size as assessed by triphenyl-tetrazolium chloride staining in pigs with an LVEF of <50% before randomization, suggesting that hyperoxemic therapy was effective in pigs with larger risk zones.

Finally, in a porcine model of 60-minute coronary artery occlusion followed by reperfusion in which SSO_2_ was evaluated for deleterious effects after primary PCI with stent implantation, Kaluza et al (48) demonstrated that 90 minutes of intracoronary SSO_2_ infusion (Pao_2_ 760-1,000 mm Hg vs 150-200 mm Hg in the normoxemic controls) did not cause coronary thrombi or affect pathology of the coronary artery at the site of stent implantation compared with normoxemic (sham) controls. Histologic analysis of the infarcted ventricles revealed that there was a nonsignificant trend toward less myocardial scarring at 30 days in the SSO_2_ group versus normoxemic controls. Echocardiography showed LV function improvement in the SSO_2_ group. The investigators concluded that SSO_2_ infusion did not cause coronary artery thrombus or toxicity to the coronary arteries or myocardium after coronary stenting in the AMI setting.

## SSO_2_ Oxygen Therapy: Clinical Studies

Preclinical studies established the safety and effectiveness of SSO_2_, and a series of clinical studies subsequently commenced. These included a phase 1/1A feasibility study, the AMIHOT I and II studies, the Optimized SSO_2_ Pilot, and the IC-HOT (Intracoronary Hyperoxemic Oxygen Therapy) study.

The feasibility study was a multicenter study of 29 patients with acute STEMI reperfused with primary PCI in which hyperoxemic blood (Pao_2_ of 600-800 mm Hg) was infused for 60 to 90 minutes after establishing proximal patency (49). Hyperoxemic reperfusion was carried out successfully in all patients, and there were no therapy-related adverse events. Echocardiographic data showed an improvement in wall motion score index and a trend toward an increase in mean LVEF at 24 hours (48.6% immediately after angioplasty vs 51.8% at 24 hours). Wall motion score and LVEF continued to progressively improve at 1 month (LVEF 54.4%) and 3 months (LVEF 56%). The major conclusion from this pilot study was that SSO_2_ therapy was safe and well-tolerated.

The AMIHOT I study was a prospective, multicenter study of 269 acute STEMI patients with either anterior or large inferior infarctions who were randomized after successful primary PCI to either 90 minutes of intracoronary hyperoxemic reperfusion using SSO_2_ therapy or normoxemic autoperfusion (50). This study enrolled subjects with up to 24 hours total ischemic time. Infarct size was measured using Tc 99m sestamibi single-photon emission computed tomographic (SPECT) imaging that was analyzed at the Mayo Clinic SPECT core laboratory. Primary endpoints were infarct size at 14-21 days, ST-segment resolution, and change in regional wall motion score index (by echocardiography) of the infarct area at 3 months post-infarction. Infarct size was 13% of the LV in the control group and 11% in the SSO_2_ group *(P =* 0.30). However, in subgroup analysis, patients <59.5 years of age, those having reperfusion time of <6 hours, and those having initial TIMI flow grade 2 demonstrated smaller infarct size with SSO_2_ *(P =* 0.02 to 0.06). In patients with anterior MIs treated within 6 hours, LV infarct size in the control group was 23% versus 9% in the hyperoxemic group *(P =* 0.04). Although change in regional wall motion score index did not differ between groups in all patients, in the subgroup of patients with anterior infarcts treated within 6 hours, the improvement in regional wall motion score was better in the SSO_2_ group versus the normoxemic group (−0.75 vs −0.54; *P =* 0.03). The area under the ST-segment deviation time curve over 3 hours did not differ among patient groups. In the subgroup of patients with anterior wall infarcts and reperfusion within 6 hours, SSO_2_ demonstrated superior reperfusion stability as assessed by serial ST segment measurements.

Although not powered for clinical events, the primary composite safety endpoint of death, reinfarction, target vessel revascularization (TVR), or stroke occurred in 5.2% of the control normoxemia group and 6.7% of the hyperoxemia group *(P =* 0.62).

As part of the AMIHOT I trial, myocardial contrast echocardiography was performed at 24 hours and at 1 month, and was reported in a substudy (51). LV end-diastolic volumes increased in the control group from 24 hours (114 mL) to 1 month (130 mL; *P <* 0.05), whereas in the SSO_2_ group, there was no increase from 24 hours (120 mL) to 1 month (117 mL). LV end-systolic volumes also increased significantly in the control group, but not the SSO_2_ group. Furthermore, LVEF increased in the SSO_2_ group from 24 hours to 1 month (49% to 54%; *P* < 0.05), whereas LVEF did not change in the control group from 24 hours (51%) to 1 month (50%). The investigators concluded that SSO_2_ in the AMIHOT I trial prevented adverse LV remodeling (LV dilatation) and improved LV ejection fraction. These findings are notable because adverse post-infarction LV remodeling is associated with worse early and late clinical outcomes (52,53).

From AMIHOT I, the investigators concluded that intracoronary hyperoxemic reperfusion with SSO_2_ therapy was safe and well-tolerated, but in the overall population did not reduce infarct size, improve regional wall motion abnormalities, or hasten ST-segment resolution on the electrocardiogram. However, in the subgroup of patients with anterior wall infarcts that were reperfused within 6 hours, hyperoxemic reperfusion appeared to provide benefit in reducing infarct size, improving regional wall motion, and stabilizing ST-segment resolution. These observations served as the rationale for AMIHOT II.

**Figure 7:**
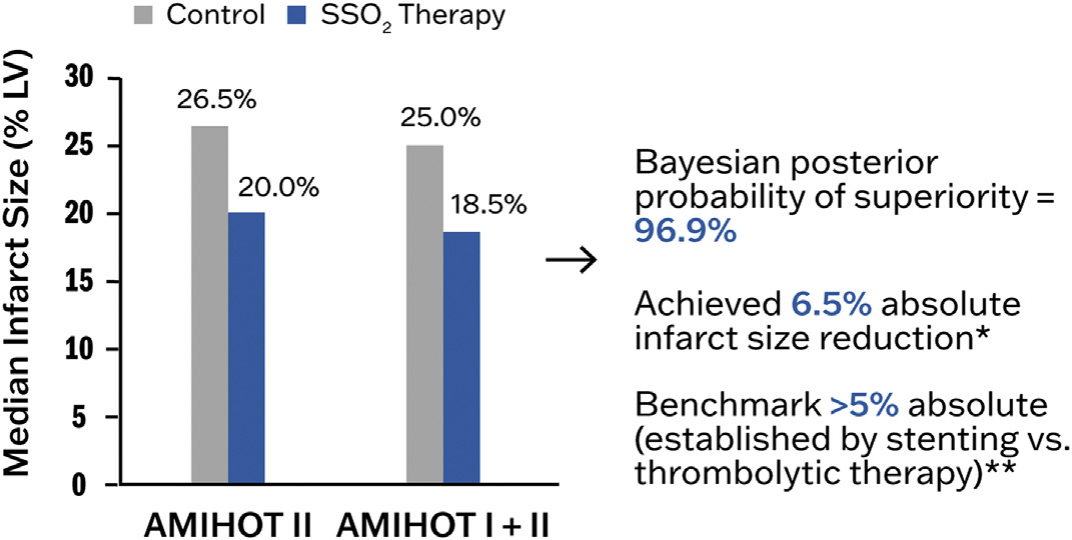
Infarct Size Results From AMIHOT II and AMIHOT I + II Infarct size results (% LV mass) from AMIHOT II and a combination of AMIHOT l and II are shown for anterior myocardial infarctions (MIs) presenting <6 hours. Overall, SSO_2_ reduced infarct size by 6.5% (absolute) compared with normoxemic reperfusion. ∗, ∗∗See Stone et al (40). AMIHOT = Acute Myocardial Infarction With Hyperoxemic Therapy study; other abbreviations as in Figure 2.

Applying the lessons learned from AMIHOT I, the AMIHOT II trial enrolled 301 patients with anterior STEMI who were reperfused by PCI with stenting within 6 hours and had achieved TIMI flow grade 2-3. Eligible patients were randomized to a 90-minute infusion of SSO_2_ therapy into the infarct artery just proximal to the implanted stent via an infusion catheter versus control (standard of care without infusion). The primary efficiency endpoint was MI size measured by nuclear scanning (Tc-99m-sestamibi SPECT) at 14 days, powered for superiority, with a safety endpoint consisting of the composite of major adverse cardiovascular events (death, reinfarction, TVR, or stroke) at 30 days, powered for noninferiority. A Bayesian hierarchical modeling technique was used to allow conditional borrowing of evidence from the AMIHOT I trial (40). Overall, 222 patients were randomized to receive SSO_2_ therapy, and 79 were randomized to control. In the AMIHOT II patients, the median infarct size in the control group was 26.5% of the LV, and in the treated group was 20.0% (unadjusted *P =* 0.10, baseline-level adjusted *P =* 0.03) (40). The pooled study-level adjusted infarct size from the AMIHOT I plus II trial analysis was 25.0% in the controls and 18.5% in the SSO_2_ therapy groups *(P =* 0.02; Bayesian posterior probability of superiority = 96.9%), meeting the prespecified superiority criteria for an absolute reduction in infarct size of 5% (40) (Figure 7). The infarct size reduction was more marked in those patients who had a baseline LVEF (on index procedure LV angiography) of <40%. In these patients, infarct size was 33.5% in controls versus 23.5% with SSO_2_, an absolute infarct size reduction of 10%.

As regards safety, there was no difference in 30-day major adverse cardiovascular events between the control (5.2%) and the SSO_2_ (6.7%) groups (posterior probability of noninferiority = 99.5% with a prospective noninferiority margin = 6%). The investigators thus concluded that in patients with anterior STEMI having successful PCI within 6 hours of symptom onset, a post-PCI 90-minute infusion of SSO_2_ therapy safely reduced infarct size. However, some adverse safety signals were evident. There was an increase in access site-related events, mainly hematomas in the SSO_2_ group (6% vs 3%). This was attributed to use of a larger infusion catheter and dual femoral access during the early phase of the study, but during the later phase of the study, hematomas were reduced by introduction of a lower profile infusion catheter and single femoral access. Stent thrombosis within 30 days occurred in 2.5% of control patients and 4.1% of SSO_2_ patients *(P =* 0.73), and incidence of mortality was 0% versus 1.8% of patients respectively *(P =* 0.58). Although the rates of stent thrombosis were not significantly different between the groups, concerns were raised that the intracoronary positioning of a catheter for SSO_2_ delivery for 90 minutes may have contributed to the observed numerical increase in thrombotic events. Specifically, contact of the distal catheter tip with the coronary luminal surface and/or flow stasis proximal to the point of infusion may have predisposed to thrombus formation, particularly if the anticoagulation regimen was subtherapeutic.

To enhance the safety of SSO_2_ therapy, an “optimized” method was developed whereby hyperoxemic blood was infused through a standard diagnostic catheter in the left main coronary artery (LM) rather than through an infusion catheter in the LAD. A 7-F femoral sheath was used for the draw line and coaxial insertion of the delivery catheter, the SSO_2_ therapy was infused through a 5-F coronary diagnostic catheter into the ostium of the LM, and the infusion time was reduced from 90 to 60 minutes by increasing the flow rate from 75 mL/min to 100 mL/min to ensure the same dose of SSO_2_ solution. Of note, a simple LM stem SSO_2_ infusion approach for 60 minutes only was used in the first 9 patients in the TherOx Phase I trial (49), with preclinical efficacy having been demonstrated with this approach in the canine model (44). Following AMIHOT II, a formal pilot study was performed with this optimized technique in 20 patients with acute anterior STEMI presenting within 6 hours of symptoms. The primary safety endpoint of target vessel failure occurred in only 1 patient (5%) within 30 days caused by stent thrombosis with reinfarction. No hemorrhagic complications were reported. The median infarct size assessed by cardiac magnetic resonance (CMR) was 13.7% at 3-5 days and 9.6% at 30 days. Mean myocardial salvage index at 3-5 days was 50%. The investigators concluded that in patients with anterior STEMI who underwent successful primary PCI, delivery of SSO_2_ for 60 minutes through a 5 Fr diagnostic catheter in the ostium of the LM was feasible, appeared safe, and resulted in a small infarct size (54).

Following this pilot study, the IC-HOT study was performed with guidance from the FDA to afford approval of SSO_2_ therapy. In this prospective, open-label, single-arm study in 100 patients, a 60-minute infusion of SSO_2_ therapy was administered to the LM through a 5-F diagnostic catheter in patients with anterior STEMIs presenting within 6 hours of symptoms after reperfusion with stenting in the proximal or mid-LAD (55). The primary safety endpoint was net adverse clinical events (NACE), the composite of death, reinfarction, TVR, or TIMI major or minor bleeding, compared with an objective performance goal of 10.7% (established from a historic control population of 112 subjects from the INFUSE-AMI [INFUSE - Anterior Myocardial Infarction (AMI)] study with similar inclusion and exclusion criteria) (56).

Infusion of SSO_2_ therapy was technically successful in 98% of patients. The rate of NACE at 30 days was 7.1%, which was lower than the 10.7% objective performance goal and also less than the 13.1% NACE rate of comparably treated patients in the AMIHOT II study. There were no deaths. There was 1 case of stent thrombosis and 1 case of severe HF. Four patients had TIMI minor bleeding with no observed incidence of serious bleeding. Infarct size, expressed as a percentage of LV mass by CMR imaging was 24.1% at 4 days and 19.4% at 30 days. Of note, LV volumes assessed by CMR fell from day 4 to day 30. The investigators concluded that the optimized infusion protocol of SSO_2_ therapy after successful primary PCI for acute anterior STEMI was feasible and had a favorable safety profile.

**Figure 8:**
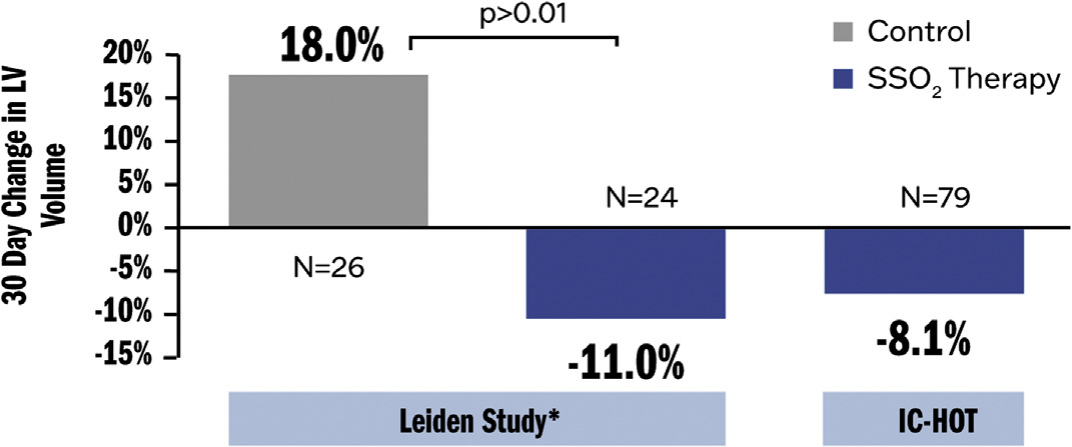
SSO_2_ and LV Dilatation SSO_2_ reduced LV dilatation (a key element of adverse LV remodeling) in both an analysis from the AMIHOT I study (Leiden substudy) as well as the IC-HOT trial. ∗See Warda et al (51). IC-HOT = Intracoronary Hyperoxemic Oxygen Therapy study; other abbreviations as in Figures 2 and 7.

The 1-year clinical outcomes from IC-HOT were compared with a propensity-matched control group of similar patients from the INFUSE-AMI study (56) (Table 2). The primary outcome of 1-year all-cause death or new-onset HF or HF hospitalizations occurred in none of the SSO_2_ therapy patients and in 12.3% of the control INFUSE-AMI patients *(P* = 0.001). SSO_2_ therapy was associated with both a lower rate of cardiovascular mortality (0% vs 4.0%; *P* = 0.04) and new-onset HF or HF hospitalizations (0% vs 7.4%; *P* = 0.01). There were no differences between groups in rates of reinfarction or TVR. Stent thrombosis within 1 year occurred in only 1 patient after SSO_2_ versus 4 control patients (1.2% vs 4.9%, respectively; *P* = 0.17). Subanalysis of the IC-HOT trial also showed similar reductions in LV volumes measured over 30 days as in the 2005 AMIHOT I substudy by Warda et al (51), confirming that early SSO_2_ therapy reduces adverse LV dilatation (remodeling) during the healing phase of infarction (Figure 8). These data suggest that infusion of SSO_2_ therapy following primary PCI in patients with anterior STEMI is safe and may improve 1-year clinical outcomes (57), although a randomized trial adequately powered for clinical events is required to determine the long-term consequences of this therapy.

**Table 2:** Outcomes at 1 Year in 166 Propensity-Score–Matched Patients From IC-HOT and INFUSE-AMI Trials

Event	IC-HOT(n = 83)	INFUSE-AMI (n = 83)	HR (95% CI)	*P* Value
Death or heart failure	0.0	12.3	—	0.001
Death	0.0	7.6	—	0.01
Heart failure	0.0	7.4	—	0.01
Reinfarction	0.0	2.4	0.97 (0.14-6.88)	0.97
TVR	2.4	5.1	0.49 (0.09-2.69)	0.40
Stent thrombosis	1.2	4.9	0.25 (0.03-2.20)	0.17
Death, heart failure, reinfarction, or TVR	3.1	14.8	0.16 (0.04-0.70)	0.005

Values are % unless otherwise indicated. IC-HOT = Intracoronary Hyperoxemic Oxygen Therapy study; INFUSE-AMI = The INFUSE - Anterior Myocardial Infarction (AMI) study; TVR = target vessel revascularization.

Details on how to perform SSO_2_ delivery and other practical issues are contained in the Supplemental Appendix.

## Conclusions

The clinical studies completed to date have demonstrated that SSO_2_ therapy is a safe and efficacious adjunctive agent to use with reperfusion to reduce infarct size, preserve cardiac function, reduce adverse LV remodeling, and potentially improve clinical outcomes in patients with acute anterior STEMI reperfused within 6 hours of symptoms. A major advantage of this treatment compared with many other investigative therapies to reduce infarct size is that reperfusion is not delayed. Future studies will examine the real-world outcomes of SSO_2_ therapy in STEMI and further explore its effect on clinical outcomes following STEMI.

## Funding Support and Author Disclosures

Drs Kloner and Burkhoff have been consultants for ZOLL TherOx. Dr Creech is an employee of ZOLL TherOx. Drs. Stone and O’Neill have been consultants for ZOLL TherOx; and were involved in previous clinical trials with SSO_2_. Dr Spears was involved with preclinical studies with SSO_2_.
