# Supplementary material for: Update on Cardioprotective Strategies for STEMI: Focus on Supersaturated Oxygen Delivery
Source: JACC Basic Transl Sci. 2021 Oct 27;6(12):1021–33. doi: 10.1016/j.jacbts.2021.07.011 (PMC8733677; doi:10.1016/j.jacbts.2021.07.011)
Supplement: Supplementary Data [file mmc1.pdf]

Electronic Supplement to: Kloner et al. Update on Cardioprotective Strategies for STEMI: Focus on Supersaturated Oxygen Delivery

Patient selection

Patient selection for the SSO_2 procedure should follow the principal inclusion and exclusion criteria from the AMIHOT II and IC-HOT trials. That is, patients should have presented within 6 hours of symptom onset with ≥1 mm ST-segment elevation in ≥2 contiguous leads in V1–V4 or new left bundle branch block and have undergone successful PCI with stent implantation to treat one or more proximal and/or mid LAD lesions with achievement of TIMI grade 2 or 3 flow. Multivessel coronary disease is not a contraindication. The extent to which patients with prior LAD infarctions may derive significant benefit is uncertain, and use in such patients should be avoided unless the prior infarct was known to be small. At the present time patients with non-LAD infarcts and those with cardiogenic shock should not be treated with SSO_2 as the safety and effectiveness of the procedure have not been demonstrated in such patients. It is likely that appropriate patient selection for SSO_2 will evolve with insights gained from post-approval registries and randomized trials, including in cardiogenic shock.

How to perform SSO_2 coronary delivery

The components of the SSO_2 delivery system consist of a polyvinyl chloride (PVC) tubing (several feet long) with a luer connection for *withdrawal* of arterial blood into the cassette; the SSO_2 Console/disposable plastic cassette placed in the computer-controlled console for preparing

the online preparation of SSO_2; the *delivery* PVC tubing (several feet long) from the cassette with a distal luer connection; and a coronary delivery catheter (See Figure 1).

Procedurally, SSO_2 coronary delivery for the interventionalist is intuitive and straightforward and is performed in the cardiac catheterization laboratory *after* successful primary PCI. A Boston Scientific #5 French JL diagnostic Impulse catheter (over a range of common shapes) is advanced through the sheath that was used to perform the PCI/stent procedure (either radial or femoral artery approach) to the left mainstem ostium. This is used for SSO_2 delivery. A bolus of contrast medium is performed to confirm that a proper position has been achieved, with 50% or more of the contrast opacifying the left anterior descending coronary artery. An arterial/withdrawal port consists of either a #5 French sheath in either the right or left femoral artery if the stent procedure had been performed from a radial approach, or via the sidearm of a #7 French femoral arterial sheath if the interventional procedure had been performed from a femoral approach. If the femoral PCI was performed with a #6 French sheath, it is exchanged for the #7 sheath to allow adequate space between the #5 French catheter and the sheath for blood withdrawal without resistance.

Either during or before reperfusion, a nurse or cardiovascular (CV) technician familiar with the TherOx Downstream System activates the TherOx Console. A clinical sterile bag of normal saline is attached to the system, The TherOx Cassette is removed from its sterile plastic bag and placed into the Console by the nurse/CV technician. The withdrawal tubing from the cassette is handed over in the sterile field to the interventionalist to attach the luer connection to the side arm of the arterial withdrawal sheath. Following computer prompts on the console, once arterial blood has been withdrawn through the cassette, the interventionalist then needs only to connect the delivery tubing to the proximal end of the #5 French Impulse catheter and perform a "wet to

wet" connection between the backflow from the catheter and the antegrade SSO_2 flow. After the connection is made, SSO_2 will flow under automated control at 100 mL/min into the left mainstem artery. Controls have been incorporated into the System so that SSO_2 flow will stop in the event that even mild hydrostatic resistance to either withdrawal or delivery is encountered. In addition, a highly sensitive microbubble ultrasonic detector on the delivery tubing will automatically trigger a shut down if >10 microliters of gas volume is detected, including summation of microbubbles to this level over a 20 minute period.

During the procedure, anticoagulation is typically achieved with systemic heparinization, with a target ACT of 300-350 secs. Blood samples just before and at 30 mins of SSO_2 are used to adjust systemic heparin dosage. Alternatively, bivalirudin anticoagulation may be used with the initial ACT >250 seconds and the bivalirudin infusion maintained at therapeutic doses without the need for further ACT checks. Oral antiplatelet loading is usually given prior to SSO_2 delivery. Glycoprotein IIb/IIIa inhibitors or cangrelor are used at the discretion of the interventionalist. Moderate sedation is used throughout the procedure and vitals are checked at 10 min intervals. Occasionally fluoroscopy is suggested to ensure that the SSO_2 diagnostic delivery catheter is moving in sync with the cardiac motion. If not, the infusion should be paused, the catheter re-positioned (verified with contrast injection), and the SSO_2 infusion re-initiated. The console warns when SSO_2 is close to completion. Once the 60-minute period of SSO_2 has been completed, an "END PROCEDURE" button on the console appears, and when pushed, allows termination of SSO_2 flow. The withdrawal and delivery tubings from the Console/cassette are disconnected manually at their Luer connections, and the sidearm Luer stem is closed on the withdrawal side. The tubings and cassette are discarded (including the small, 75 mL priming blood volume within the SSO_2 extracorporeal circuit). The #5 French Impulse catheter is

connected to the multiport Luer manifold, and if desired, the interventionalist at his or her discretion can inject contrast medium to confirm catheter position and coronary flow in the LAD. The catheter is removed as are the sheaths as per standard protocols. Moderate sedation is stopped, and the patient then is transferred to a suitable monitoring unit.

Limitations in introducing SSO_2 into practice

1) Concern about lack of evidence of clinical benefit. Infarct size was, appropriately, the primary effectiveness endpoint in the AMIHOT trials, as it is an objective, highly validated measure that strongly predicts the subsequent risk of death and heart failure in STEMI patients (1). Moreover, in several sub-studies of clinical SSO_2 trials that examined functional recovery, significant improvements in left ventricular function were found at follow-up (2,3). Nevertheless, the AMIHOT trials were not powered for improvement in clinical outcomes, although such a benefit was reported in the retrospective comparison of the IC-HOT outcomes compared to a propensity-adjusted control population from INFUSE-AMI (4). A post-approval randomized trial (AMIHOT III) is planned in 434 pts with non-shock anterior STEMI and successful primary PCI (TIMI 2 or 3 flow) within 6 hours of symptom onset at 60 US/non-US sites (5) and when combined with AMIHOT I and II may provide adequate data to demonstrate clinical evidence of improved outcomes after SSO_2 infusion after primary PCI in anterior STEMI.

2) Confusion as to risk vs. benefits of high O_2 levels in STEMI. The concept of lethal reperfusion injury, caused primarily by reactive oxygen species, has dominated research on infarct size reduction for decades. In addition, the oxidative stress theory of disease in general has been studied intensively since the original concepts were published in the 1950's (6,7). As

discussed by Ghezzi et al (8), despite many decades of research in many different medical specialties, "…no antioxidant has been approved as therapy by any regulatory agency in countries that base their decisions on evidence-based medicine."

More recently, the "AVOID Trial" received a great deal of media attention related to potential adverse effects of high flow oxygen in acute coronary syndrome patients who were not hypoxic (9). However, subsequent similar but much larger trials demonstrated no effects (neither adverse nor beneficial) of high flow oxygen on any clinical endpoint (10,11). These studies received little media attention, but the acronym (DETOX2-AMI) for the most definitive trial (9) may have mislead many readers as to the true results of the trial (that is, oxygen was *not* toxic).

Conversely, the lack of clinical recognition of reperfusion microvascular ischemia (RMI), a term coined by one of us (JRS), in the pathogenesis of infarct size expansion has received little attention because of difficulties in its detection, quantitation, and imaging as discussed by Spears JR (12).

3) Practical issues. The need to observe the patient in the catheterization lab for one hour beyond successful reperfusion has been an impediment to clinical acceptance, especially for infarcts occurring between 12 PM and 6 AM. One rationale for the use of the sub-selective intracoronary perfusion tubing in the AMIHOT I and II trials was to enable transfer of the patient from the cath lab to a clinical monitoring unit, without concern for dislodgment of the catheter. This was particularly helpful, when SSO_2 infusion was performed for 90 min. However, as discussed earlier herein, the presence of the intracoronary catheter may have increased the risk of stent thrombosis. The current "Optimized" SSO_2 delivery system, with the #5 French catheter tip positioned in the ostium of the left mainstem coronary, requires that the patient remain stationary

in the cath lab. However, the shorter, 60 min of SSO_2 perfusion is more practical, without reducing the level of infarct size reduction (13).

Other practical issues include the upfront cost of the downstream system console, individual cassettes and other equipment, a consideration that will hopefully be mitigated with improved reimbursement.

References

1. Stone GW, Selker HP, Thiele H et al. Relationship Between Infarct Size and Outcomes Following Primary PCI: Patient-Level Analysis From 10 Randomized Trials. J Am Coll Cardiol 2016;67:1674-83.

2. Dixon SR, Bartorelli AL, Marcovitz PA , Spears R, David S, Grinberg I, Qureshi MA, Pepi M, Trabattoni D, Fabbiocchi F et al. Initial experience with hyperoxemic reperfusion after primary angioplasty for acute myocardial infarction: results of a pilot study utilizing intracoronary aqueous oxygen therapy. J Am Coll Cardiol 2002;39:387-92.
3. Trabattoni D, Bartorelli AL, Fabbiocchi F, Montorsi P, Ravagnani P, Pepi M, Celeste F, Maltagliati A, Marenzi G, O'Neill WW. Hyperoxemic perfusion of the left anterior descending coronary artery after primary angioplasty in anterior ST-elevation myocardial infarction. Catheter Cardiocasc Interv. 2006; 67: 859-65.
4. Chen S, David SW, Khan ZA et al. One-year outcomes of supersaturated oxygen therapy in acute anterior myocardial infarction: The IC-HOT study. Catheter Cardiovasc Interv 2021;97:1120-1126
5. ClinicalTrials.gov Identifier: NCT04743245.

6. Gerschman R, Gilbert D, Nye SW, Dwyer P, Fenn WO. Oxygen Poisoning and X-irradiation: a Mechanism in Common. Science 1954. Vol 119: 623-626. DOI: 10.1126/science.119.3097.623

7. Harman D. Aging: a theory based on free radical and radiation chemistry. J Gerontology. 1956. Vol 11: 298-300. https://doi.org/10.1093/geronj/11.3.298

8. Ghezzi P, Jaquet V, Marcucci F, Schmidt HHHW. Review Article. The oxidative stress theory of disease: levels of evidence and epistemological aspects. Brit J Pharm. 2017. Vol 174: 1784-1796. DOI:10.1111/bph.13544

9. Stub D, Smith K, Bernard S, Nehme Z, Stephenson M, Bray JE, Cameron P, Barger B, Ellims AH, Taylor AJ et al. Air versus oxygen in ST-segment-elevation myocardial infarction. Circulation 2015; 131:2143-2150.

10. Hofmann R, James SK, Jernberg T, Lindahl B, Erlinge D, Witt N, Arefalk G, Frick M, Alfredsson J, Nilsson L et al. Effect of oxygen therapy in suspected acute myocardial infarction. N Engl J Med 2017;377:1240-1249. doi: 10.1056/NEJMoa1706222

11. Sepehrvand N, James SK, Stub D, Khoshnood A, Ezekowitz JA, Hofmann R. Effects of supplemental oxygen therapy in patients with suspected acute myocardial infarction: a meta-analysis of randomized clinical trials. Heart 2018;104:1691-1698. doi: 10.1136/heartjnl-2018-313089

12. Spears JR. Reperfusion microvascular ischemia after prolonged coronary occlusion: implications and treatment with local SSO2 delivery. Hypoxia 2019; 7;65-79. doi.org/10.2147/HP.S217955

13. Spears JR, Henney C, Prcevski P , Xu R, Li L, Breneton GJ, DiCarli M, Spanta A, Crilly R, Sulaiman AM et al.. Aqueous oxygen hyperbaric reperfusion in a porcine model of myocardial infarction. J Invasive Cardiol 2002;14:160-6.
